# Effects of peer health education on perception and practice of screening for cervical cancer among urban residential women in south-east Nigeria: a before and after study

**DOI:** 10.1186/s12905-017-0399-6

**Published:** 2017-06-09

**Authors:** Chinyere Mbachu, Cyril Dim, Uche Ezeoke

**Affiliations:** 1 Department of Pharmacology and Therapeutics, Health Policy Research Group, College of Medicine, University of Nigeria, Nsukka, Nigeria; 2 Department of Obstetrics and Gynecology, College of Medicine, University of Nigeria, Nsukka, Nigeria; 3 Department of Community Medicine, College of Medicine, University of Nigeria, Nsukka, Nigeria

**Keywords:** Cervical cancer, Screening, Peer education, Perception, Practice

## Abstract

**Background:**

Effective female education on cervical cancer prevention has been shown to increase awareness and uptake of screening. However, sustaining increase in uptake poses a challenge to control efforts. Peer health education has been used as an effective tool for ensuring sustained behavior change. This study was undertaken to assess the effectiveness of peer health education on perception, willingness to screen and uptake of cervical cancer screening by women.

**Methods:**

A before and after intervention study was undertaken in 2 urban cities in Enugu state, Nigeria among women of reproductive age attending women’s meeting in Anglican churches. Multistage sampling was used to select 300 women. Peer health education was provided once monthly for 3 consecutive sessions over a period of 3 months. Data was collected at baseline and after the intervention using pre-tested questionnaires. Descriptive statistics and tests of significance of observed differences and associations were done at *p*-value of <0.05.

**Results:**

Statistical significant difference was observed in participants’ individual risk perception for cervical cancer and perception of benefits of early detection through screening. Practice of screening for cervical cancer increased by 6.8% and the observed difference was statistically significant (*p* = 0.02). This was significantly associated with marital status, level of education, employment status and parity (*p* < 0.05).

**Conclusion:**

Peer health education is an effective strategy for increasing women’s perception of benefits of early detection of cervical cancer through screening. It is also effective for increasing their practice of screening for cervical cancer.

**Electronic supplementary material:**

The online version of this article (doi:10.1186/s12905-017-0399-6) contains supplementary material, which is available to authorized users.

## Background

Cervical cancer is the most common cancer among women in sub-Saharan Africa and its incidence and mortality are on the increase [1]. It ranks as the second most common cancer among women in Nigeria, contributing an estimated 14,089 (21.8%) cancer cases and 8240 (20.3%) cancer deaths in 2012 [2, 3]. Every year, 14,550 Nigerian women are diagnosed with cervical cancer, out of which 9659 die from the disease. It is projected that by 2025 an estimated 22,914 women would develop cervical cancer annually, out of which 16,261 would die [1].

Cervical cancer provides unique opportunities for prevention because central to its etiology is infection of the cervix with a well-defined virus, the human papilloma virus [4, 5]. The process of developing cervical cancer following infection takes at least 10 to 20 years, allowing time to intervene long before cancer develops [6]. The existence of effective cervical cancer control programs, and the frequency of screening account for the observed difference in cervical cancer mortality between developed and developing countries [7]. There are several screening tests for cervical cancer such as conventional cytology (Pap smear), liquid-based cytology, visual inspection with acetic acid (VIA) and Lugol’s iodine (VILI), and Human papilloma virus (HPV) DNA test [8]. Regular screening will detect pre-cancerous changes and reduce the risk of developing cervical cancer. In sub-Saharan Africa however, there are few organized efforts to ensure that women undergo screening for early detection of cervical cancer and reduction in morbidity and mortality [9].

The level of uptake of cervical cancer screening in Nigerian women is abysmally low and unrelated to levels of awareness [10–15]. A study among young women in a Nigerian tertiary institution found an awareness level of over 60% and a screening rate of 0% [16]. Among health professionals (nurses) in South-east Nigeria, an awareness level of 87% for screening services was reported, with an uptake of 5.7% [17]. Even where cervical cancer screening services are available, they are being underutilized [16, 18, 19]; as is the case in Enugu state where over a period of ten years, the level of participation was <1% of the targeted population [12]. Wrong perceptions and fear of the unknown are some of the reasons that have been given for under-utilization of cervical cancer screening services in Nigeria [13, 16, 17].

Effective female education on cervical cancer and screening, among other strategies, has been identified as a method of increasing awareness and utilization of cervical cancer screening services [10, 11, 13, 20]. Since health education also involves “provision of information to address the underlying social, economic and environmental conditions impacting on health”, we need to develop and adopt strategies that are sensitive and relevant to social contexts [21]. The relevance of peer education to health lies in its ability to address the social under-lays that impact on health [21]. Peer education draws on the Diffusion of Innovation Behavioral Theory which states that “certain individuals from a given population act as agents of change by disseminating information and influencing group norms in their community” [22]. The effectiveness of peer education/lay health worker programs in public health has been documented in immunization uptake, nutrition education, family planning, and HIV prevention, care and support [22, 23]. However, there is very limited local evidence of the effects of context relevant health education interventions on cervical cancer control.

This study was undertaken to examine whether peer health education would influence women’s perception of cervical cancer and its screening and their practice of screening for the disease. This paper provides new knowledge on the effectiveness of a context-appropriate peer health education intervention on perception and practice of screening for cervical cancer in urban areas in Nigeria.

## Methods

### Study design and setting

This was a community-based intervention study using a before and after design.

This study was conducted in Enugu state, located in the south east geopolitical zone of Nigeria. The 2006 population census estimates a total of 5,590,513 (2005 estimate) inhabitants within an area of 7618 sq. km [24]. The State has three urban centers namely: Enugu, Nsukka and Oji River and at the time of the study, there were 4 Anglican dioceses in the urban centers. These urban centers are similar in terms of income, education, culture and religion.

For the purpose of health care delivery, the state is divided into seven health districts namely: Awgu, Udi, Enugu Ezike, Nsukka, Enugu Metropolis, Isi–Uzo and Agbani. Each health district serves a population of not less than 50,000 people, and it has a range of public health facilities including a district hospital, general hospitals and primary health centers. There are also privately owned hospitals, pharmacies, laboratories and patent medicine shops that serve as important sources of health service delivery. The state also has four tertiary hospitals, three in Enugu metropolis and one in Agbani district [25].

Pap smear cytology for cervical cancer screening is provided at varying costs in some of these health facilities and at the time of the study it was being offered in four health facilities – two within Enugu metropolis, one in Agbani district and a private hospital in Nsukka district. The availability of Pap smear cytology services informed the selection of Enugu and Nsukka urban centres for the study. The study was conducted in the Anglican dioceses of Enugu and Nsukka.

### Description of study population and participant characteristics

The study population consisted of women of reproductive age who resided in the study area and worshipped in the selected Anglican dioceses. Only women who were 21 years or more; currently sexually active or have been in the past; and never been diagnosed with any cancer, were eligible for the study. Screening before 21 years may lead to unnecessary treatment of resolvable pre-invasive lesions and increased risk of reproductive harm [26]. Cervical changes preceding cancer are most significant after 21 years and that is the age at which screening becomes relevant [26].

The peer health educators were wives of clergy men in the dioceses who had completed secondary school education and volunteered to be trained. These group of women are role models because they are perceived to have high moral standards. They lead women groups in the churches and are well respected in the community for the important position they occupy in the church. While it is encouraged that women obey instructions given by the clergy wives, they are not under any obligation to do so. They elect their executives and clergy wives are members. Clergy wives do not have overriding decision making power.

### Sampling of study participants

In order to observe a difference of 13% in the practice of cervical cancer screening at a power of 80% and confidence level of 95%, a minimum sample of 270 respondents was needed. The sample size was increased to 300 to adjust for incomplete and non-response. This difference of 13% is an average that was taken from review of literature that showed an increase in cervical cancer screening of 10% to 15% following health education interventions [27, 28].

Multistage sampling technique was used. Two of the four urban dioceses were selected by simple random sampling at the first stage. At the second stage, 6 parishes were selected by balloting without replacement from a list of all the parishes in the diocese. Equal numbers of women (50 each) were consecutively recruited from each of the five parishes at the final stage, to give 300 participants.

All clergy wives in selected parishes were invited to a meeting where they were informed about the study, their expected roles and reward. A request was then made for volunteers who will be trained as peer health educators. The volunteers were not paid or given any honorarium. They were only reimbursed for their transport expenses during the training period.

### Description of Intervention

For the peer health educators’ training, 22 volunteers were recruited and trained from among clergy wives to provide peer health education on cervical cancer and its prevention to women in their parishes. An 18-h peer educators’ training workshop was provided over a three-day period using the peer educators’ training manual of the Cervical Cancer Prevention Program in Zambia (CCPPZ), which was adapted for the study and context [29]. The adaptations included burden of cervical cancer in Nigeria, use of local names for cancer and other concepts, and non-inclusion of some sections such as the section on HIV/AIDS, and integrating HIV testing with cervical cancer screening.

After the training, the peer health educators provided repeated sessions of health education on cervical cancer and its prevention to women in their parishes during monthly meetings for at least 3 consecutive months. In this period, the women were exposed to cervical cancer prevention peer education for a minimum of 6 times, and respondents attended 3 to 6 sessions. In all parishes, women met at least two times a month during the monthly general meeting and union meetings. These are established and regular meetings that all women are expected to attend and during which peer education on cervical cancer screening was provided. The peer educators were encouraged to use other opportunities that present to provide health education on cervical cancer prevention. However, these sessions were not considered for eligibility in this study.

The peer health education intervention commenced in the next meeting after baseline data was collected. Each group session consisted of 45–60 min of didactic teaching on what the cervix is and how it can be kept healthy; definition of cervical cancer; burden of cervical cancer in Nigeria relative to other settings; risk factors, symptoms and signs of cervical cancer; prevention and treatment options for cervical cancer; and healthy living with cervical cancer. This was followed by 15–30 min of clarifications and feedback.

### Data collection and analysis

Data was collected at baseline and after the peer health education intervention using a pre-tested structured questionnaire (see Additional file 1: APPENDIX-Questionnaire.pdf). The questionnaire was self-administered and completed individually by the respondents. Clarifications to questions were provided individually to respondents. Baseline data was collected from the women during the monthly meeting that preceded their first session of cervical cancer prevention education with the peer health educators. They were informed of the study and its objectives, and told that although they could all benefit from the peer education sessions that would happen in their next meetings, only the first 50 people in attendance have been selected to answer the questionnaires. Detail of what the peer health education would cover was not provided before baseline data collection to eliminate biased perception of individual risks.

Only those who participated in at least 3 of the 6 group training sessions were eligible to be interviewed after the intervention. The meeting attendance register was used to identify those who were eligible for post-intervention data collection.

Information was collected on respondents’ socio-demographic characteristics, their perception of severity and individual risk for cervical cancer, their perception of benefits of screening for cervical cancer, their willingness to screen for cervical cancer and actual practice of screening for cervical cancer. The questionnaires were administered simultaneously to selected parishes.

Data was organized and analyzed using statistical package for social sciences (SPSS) statistics version 17. This is a statistical analysis software for managing structured and unstructured data. It includes a broad range of statistical procedures for understanding data and generating reliable results [30]. The variables of interest or outcome measures were perception of individual risk for cervical cancer and benefits of screening, willingness to screen, actual practice of screening, preferred method, frequency of screening and reasons for screening. Frequency and percent were used to describe the distribution of variables, cross-tabulation of proportions was done to check difference in cervical cancer screening before and after the intervention, and to identify factors associated with screening for cervical cancer. Chi-square test was performed to assess statistical significance of observed differences and associations. Logistic regression analysis of factors associated with screening for cervical cancer amongst women who had screened for cervical cancer [31]. Statistical significance was set at a *p*-value of <0.05.

### Ethical consideration

Ethical approval was sought for and obtained from the Health Research Ethics Committee of the University of Nigeria Teaching Hospital in Enugu (NHREC05/01/08-FWA00002458-IRB00002323). Written informed consent was obtained from the participants before the data collection tools were administered, and confidentiality was maintained by non-inclusion of self-identifying characteristics in the questionnaires. The data was aggregated for analysis.

## Results

The results of the study are presented in Tables 1, 2, 3 and 4. A total of 300 questionnaires were distributed to the 300 recruited participants before the intervention; 285 of these were appropriately filled and suitable for analysis. Some questionnaires were discarded because they were not completely filled; some non-skip questions were not answered. Some questionnaires were discarded because of disparity in recorded responses. For instance, some questionnaires had an entry on ‘No’ to screening for cervical cancer as well as an entry for type of screening method used. Following the intervention, 2 more participants could not be followed up, giving a total of 283 responses post-intervention.

**Table 1:** Socio-demographic characteristics of respondents

Variables		Frequency (%)
		(*N* = 285)
Age categories (years)	25–34	18 (6.3)
	35–44	63 (22.1)
	45–54	133 (46.7)
	>54	71 (24.9)
Marital status	Married	208 (73)
	Never married	17 (6)
	Divorced/Separated	0
	Widowed	60 (21)
	Co-habiting/Living with partner	0
Level of education	No formal education	0
	Primary school – completed	16 (5.6)
	Primary school – not completed	10 (3.5)
	Secondary school – completed	46 (16.1)
	Secondary school – not completed	13 (4.6)
	^a^Tertiary school – completed	142 (49.8)
	^a^Tertiary school – not completed	58 (20.4)
Employment status	Paid employment	199 (69.8)
	Self-employed	50 (17.5)
	Unemployed	18 (6.3)
	Student	18 (6.3)
Parity	One	30 (10.5)
	Two	29 (10.2)
	Three	31 (10.9)
	Four	74 (26.0)
	More than four	121 (42.5)

^a^Tertiary school means any formal education that happened after secondary schooling, such as University education, Schools of Technology, Schools of Catering and Hospitality Management

**Table 2:** Perception of cervical cancer and Practice of its screening

Perception variables	Pre-intervention (*N* = 285); n (%)	Post-intervention (*N* = 283); n (%)	*p*-value
Perception of severity			
Not severe	153 (53.7)	0	-
As serious as other cancers	131 (45.9)	219 (77.4)	<0.001
Less severe than other cancers	1 (0.4)	64 (22.6)	<0.001
Individual risk perception			
At risk	74 (26)	113 (40)	0.01
Not at risk	211 (74)	170 (60)	0.01
Perception of benefit of screening			
Not beneficial	98 (34.4)	33 (11.7)	0.001
Highly beneficial	145 (50.9)	195(68.9)	0.01
Slightly beneficial	42 (14.7)	55 (19.4)	0.17
Willing to screen for cervical cancer	226 (79.3)	228 (80.8)	0.52
Screened for cervical cancer	30 (10.5)	49 (17.3)	0.02
• Screening method			
✓ Pap smear test	27 (9.5)	45 (15.9)	0.02
✓ Others (VILI and VIA)	3 (1.0)	4 (1.4)	0.69
Reasons for not screening	15 (5.3)		
Fear of positive result		40 (14.1)	<0.001
Do not have symptoms	85 (29.9)	104 (36.7)	0.07
Cost of screening	12 (4.3)	24 (8.5)	0.06
Distance of screening center	37 (13)	46 (16.2)	0.3
Fear of pain	9 (3.2)	0 (0)	0.001
Don’t know screening tests	118 (41.4)	0 (0)	0.001
Don’t know where to screen	-	81 (28.6)	0.001

**Table 3:** Socio-demographic factors associated with practice of screening for cervical cancer

Socio-demographic variables	Screening rate for cervical cancer (*N* = 49) n (%)	*p*-value
Age categories (years)		
25–34	4 (8.2)	0.33
35–44	10 (20.4)	
45–54	28 (57.1)	
> 54	7 (14.3)	
Marital status		
Married	40 (81.6)	0.03
Never married/Divorced/Co-habiting	5 (10.2)	
Widowed	4 (8.2)	
Level of education		
None	-	<0.001
Primary	0 (0)	
Secondary	0 (0)	
Tertiary school	49 (100)	
Employment status		
Unemployed	5 (10.2)	<0.001
Student	1 (2)	
Employed (self- or paid-)	43 (87.8)	
Parity (number of children)		
None	1 (2.0)	<0.001
1–2	10 (20.4)	
3–4	27 (55.1)	
5 or more	11 (22.4)

**Table 4:** Regression analysis of factors associated with screening for cervical cancer post-intervention in the study group

Variables		Unstandardized coefficient	OR	*p*-value	95% C.I. for OR	
					Lower	Upper
Age category	^a^25–34			0.431		
	35–44	0.083	1.086	0.847	0.468	2.525
	45–54	−0.044	0.957	0.922	0.395	2.318
	>54	−0.870	0.419	0.167	0.122	1.440
	Constant	−1.482	0.227	0.000		
Marital status	^a^Widowed			0.042		
	Married	1.204	3.333	0.028	1.142	9.731
	Never married	1.764	5.833	0.018	1.361	24.996
	Constant	−2.639	0.071	0.000		
Level of education	^a^Primary			1.000		
	Secondary	0.000	1.000	1.000	0.000	.
	Tertiary	20.077	5.242	0.998	0.000	.
	Constant	21.203	0.000	0.998		
Employment status	^a^Unemployed			0.448		
	Student	−1.878	0.153	0.104	0.016	1.473
	Self-employed	−20.247	0.000	0.997	0.000	.
	Paid employment	−0.333	0.717	0.547	0.242	2.121
	Constant	−0.956	0.385	0.385		
Parity	^a^None			0.002		
	1–2	0.896	2.449	0.414	0.285	21.032
	3–4	1.606	4.985	0.132	0.617	40.251
	5 or more	0.182	1.200	0.867	0.142	10.119
	Constant	−2.485	0.083	0.017		

^a^Reference category

### Demographic characteristics

Results of respondents’ socio-demographic variables are presented in Table 1. The mean age of respondents was 48 (±9.7) years, and the highest proportion were in the 45–54 years’ age category. Majority of the respondents were married, 208 (73%). The highest proportions of respondents had completed tertiary education, 142 (49.8%). Majority of them were employed (paid or self): 199 (69.8%) were in paid employment, 50 (17.5%) were self-employed. Most of the respondents had four or more children, 121 (42.5%).

### Perception and practice of screening for cervical cancer

Participants’ perception and practice of screening for cervical cancer are presented in Table 2. Following the peer health education intervention, significantly higher proportions of participants, 219 (77.4%), perceived cervical cancer to be as serious as other cancers (*p* < 0.001); and themselves at risk of developing cervical cancer, 113 (40%) (*p* = 0.01). The proportion of women who felt screening for the disease would be highly beneficial increased significantly from 145 (50.9%) to 195 (68.9%) after the intervention (*p* = 0.01).

Willingness to screen for cervical cancer was relatively high at baseline, 226 (79.3%), but not much change was observed following the intervention, 228 (80.8%). There was statistical significant increase in rate of screening for cervical cancer after the intervention (*p* = 0.02). The proportion of participants who screened for cervical cancer increased from 30 (10.5%) before the intervention to 49 (17.3%) after the intervention.

### Relationship between socio-demographic characteristics and cervical cancer screening

Table 3 shows the relationship between respondents’ socio-demographics and their practice of screening for cervical cancer. There was no significant difference in practice of screening among respondents of different age categories.

There was statistical significant difference in practice of screening for cervical cancer among respondents with different marital status, levels of education, employment status and parity (*p* < 0.05). Married women were more likely to have ever screened for cervical cancer than the rest. Out of the 49respondents who screened for cervical cancer, 40 (81.6%) were married. Only those who had tertiary education had ever screened for cervical cancer, 49 (100%). Women who were employed 43 (87.8%) and had more (3–4) children were more likely to have screened for cervical cancer, 27 (55.1%).

### Logistic regression of factors associated with screening for cervical cancer

Table 4 shows regression analysis of factors associated with screening for cervical cancer amongst women who had screened for cervical cancer after the intervention. The odds of screening for cervical cancer was 3.3 times more in married women and 5.8 times more in women who had never been married than in those who were widowed (*p* < 0.05). Women who had 1–2 children were 2.4 times more likely to have screened for cervical cancer than those who did not have children; and those who had 3–4 children were 4.99 times more likely to have screened for cervical cancer than those who did not have children.

## Discussion

Although more than three-quarters of the participants expressed willingness to screen for cervical cancer, we found that relatively high proportions of them neither perceived it to be as serious as other cancers nor themselves at risk of developing or having the disease. This could explain the baseline finding of very low cervical cancer screening rate among these women. The frequent contacts women make with health services ought to provide unique opportunities for health workers to educate them on cervical cancer and its screening and to offer opportunistic screening services. However, for various reasons such as non-availability of cancer screening services and lack of expertise, this is often not the case [32–34]. Among respondents who had screened for cervical cancer, the commonest screening method used is the Pap smear test. This is probably because it is the most available and affordable method.

Women’s perception and practice of screening for cervical cancer were improved by peer health education. Having received comprehensive information on the risks, uncertainties and benefits of the screening, the women were able to assess their risk status and take necessary action. There were statistical significant differences in their perceptions of the severity of cervical cancer, their individual risks of developing the disease and the benefits of screening. Based on the relative advantage element of diffusion of innovation theory respondents’ perceptions of benefits of screening for cervical cancer changed [35]. More women perceived screening as beneficial following peer health education. Having also observed their peers now promoting cervical cancer screening, the behavior became desirable and more women adopted the behavior [35]. Although the observed change in practice was slight, a sustained increase could be expected over time if the peer health educators continue to educate the women repeatedly. The ultimate goal of reinforced health education is lasting change in behavior [36]. This and more could be achieved by peer health education for as long as the peer health educators remain in the community and continue to tell their peers about cervical cancer and the benefits of early detection through screening [37, 38].

Marital status, level of education, employment status and parity were all significantly associated with the practice of screening for cervical cancer. Women who were employed, married and of higher parity and level of education were more likely to screen for cervical cancer. Since women who are employed are earning an income, they are more likely to afford preventive health services than those who are unemployed. Compared to less educated women, more educated women have been reported to choose and utilize preventive health services, having been informed of the implications of their choices. In the same vein, married were more likely to have screened than those who were not married, for reasons such as having the social and financial support of a spouse [39]. These three factors are closely linked to affordability and explain the disparity between practice of screening for cervical cancer and expressed willingness to screen [40].

The scope for generalization of findings of this study is limited to women residing in urban and peri-urban areas and attending women’s meeting in Anglican diocese in south-east Nigeria. The study population consisted of Anglican women who belonged to the women’s fellowship, implying that some social and cultural factors that influence behavior have already been controlled and their effects cannot be measured. Equal allocation was used at the final stage of selection of participants and while this could be critiqued for representation, the number of participants selected from each parish exceeded 70% of the eligible population.

## Conclusions

Willingness to screen for cervical cancer is considerably high amongst the study participants. Peer health education is an effective strategy for increasing women’s perception of the benefits of early detection of cervical cancer and their practice of screening for it. It also has proven effectiveness in addressing the cultural and social factors that influence behavior change. Peer health education should be included in the health promotion component of cervical cancer control plans, because it could be useful for ensuring improved cervical cancer screening behavior.

## Additional file

Additional file 1: Questionnaire for data collection on knowledge, perception and practice of screening for cervical cancer. Structured questionnaire with two open-ended questions, and having three sections namely, (i) personal data, (ii) knowledge and perception of cervical cancer and cervical cancer screening, (iii) utilization of cervical cancer screening services and determinants. (PDF 337 kb)

## References

[1] GLOBOCAN. Cervical Cancer: Estimated Incidence, Mortality and Prevalence in 2012. http://globocan.iarc.fr/Pages/fact_sheets_cancer.aspx.

[2] GLOBOCAN. Nigeria: Female. Estimated number of cancer cases, all female. 2012. http://globocan.iarc.fr/old/pie_pop.asp?selection=143566&title=Nigeria&sex=2&type=0&window=1&join=1&submit=%C2%A0Execute.

[3] GLOBOCAN. Nigeria: Females. Estimated number of cancer deaths, all ages. 2012. http://globocan.iarc.fr/old/pie_pop.asp?selection=143566&title=Nigeria&sex=2&type=1&window=1&join=1&submit=%C2%A0Execute.

[4] Bosch F, Lorincz A, Munoz N, Meijer C, Shah K (2002). The causal relation between human papillomavirus and cervical cancer. J Clin Pathol.

[5] Meanwell C, Blackledge G, Cox M, Maitland N (1987). HPV 16 DNA in normal and malignant cervical epithelium: implications for the aetiology and behaviour of cervical neoplasia. Lancet.

[6] Denny L (2008). Prevention of cervical cancer. Reprod Health Matters.

[7] GAVI-UNFPA-WHO. Strengthening cervical cancer prevention and control. 2009.

[8] Health WHOR, Diseases WHOC, Promotion H. Comprehensive cervical cancer control: a guide to essential practice. Lusaka: World Health Organization; 2006. 25642554

[9] WHO/IARC/APHRC. Prevention of cervical cancer through screening using visual inspection with acetic acid (VIA) and treatment with cryotherapy. Malawi, Madagascar, Nigeria, Uganda, the United Republic of Tanzania, and Zambia: A demonstration project in six African countries; 2012.

[10] Anyebe E, Opaluwa S, Muktar H, Philip F (2014). Knowledge and Practice of Cervical Cancer Screening amongst Nurses in Ahmadu Bello University Teaching Hospital Zaria. Research on Humanities and Social Sciences.

[11] Oche M, Kaoje A, Gana G, Ango J (2013). Cancer of the cervix and cervical screening: Current knowledge, attitude and practices of female health workers in Sokoto, Nigeria. International Journal of Medicine and Medical Sciences.

[12] Chukwuali LI, Onuigbo WI, Mgbor NC (2004). Cervical Cancer Screening in Enugu, Nigeria. Trop J Obstet Gynaecol.

[13] Nwankwo KC, Aniebue UU, Aguwa EN, Anarado AN, Agunwah E (2011). Knowledge attitudes and practices of cervical cancer screening among urban and rural Nigerian women: a call for education and mass screening. Eur J Cancer Care (Engl).

[14] Ezem B (2007). Awareness and uptake of cervical cancer screening in Owerri, South-Eastern Nigeria. Ann Afr Med.

[15] Aniebue P, Aniebue U (2010). Awareness and practice of cervical cancer screening among female undergraduate students in a Nigerian university. J Cancer Educ.

[16] Akujobi CN, Ikechebelu JI, Onunkwo I, Onyiaorah IV (2008). Knowledge, attitude and practice of screening for cervical cancer among female students of a tertiary institution in South Eastern Nigeria. Niger J Clin Pract.

[17] Udigwe GO (2006). Knowledge, attitude and practice of cervical cancer screening (pap smear) among female nurses in Nnewi, South Eastern Nigeria. Niger J Clin Pract.

[18] Anorlu RI, Ribiu KA, Abudu OO, Ola ER (2007). Cervical cancer screening practices among general practitioners in Lagos Nigeria. J Obstet Gynaecol.

[19] Aboyeji PA, Ijaiya MA, Jimoh AA (2004). Knowledge, Attitude and Practice of Cervical Smear as a Screening Proceedure for Cervical Cancer in Ilorin, Nigeria. Trop J Obstet Gynaecol.

[20] Wright KO, Kuyinu YA, Faduyile FA (2010). Community education on cervical cancer amongst market women in an urban area of Lagos, Nigeria. Asian Pac J Cancer Prev.

[21] WHO. Health education: theoretical concepts, effective strategies and core competencies: a foundation document to guide capacity development of health educators**.** In*.* http://applicationsemrowhoint/dsaf/EMRPUB_2012_EN_1362pdf.; 2012.

[22] Kaminski J (2011). Diffusion of innovation theory. Canadian Journal of Nursing Informatics.

[23] Lewin S, Munabi-Babigumira S, Glenton C, Daniels K, Bosch-Capblanch X, van Wyk BE, et al. Lay health workers in primary and community health care for maternal and child health and the management of infectious diseases. Cochrane Database Syst Rev. 2010;3:CD004015. 10.1002/14651858.CD004015.pub3 PMC6485809 20238326

[24] Federal Republic of Nigeria (2007). Publication of the Details of the Breakdown of the National and State Provisional Total 2006 Census: Official Gazette.

[25] Enugu State Ministry of Health. Enugu: Health Service Delivery. 1st ed. Enugu: Planning Research Statistics Department; 2010.

[26] Saslow D, Solomon D, Lawson HW, Killackey M, Kulasingam SL, Cain J, et al. American Cancer Society, American Society for Colposcopy and Cervical Pathology, and American Society for Clinical Pathology screening guidelines for the prevention and early detection of cervical cancer. CA Cancer J Clin. 2012;62(3):147–72. 10.3322/caac.21139 PMC3801360 22422631

[27] Perkins RB, Langrish S, Stern LJ, Simon CJ (2007). A community-based education program about cervical cancer improves knowledge and screening behavior in Honduran women. Rev Panam Salud Publica.

[28] Fernández ME, Gonzales A, Tortolero-Luna G, Williams J, Saavedra-Embesi M, Chan W, et al. Effectiveness of Cultivando La Salud: A Breast and Cervical Cancer Screening Promotion Program for Low-Income Hispanic Women. Am J Public Health. 2009;99(5):936–43. 10.2105/AJPH.2008.136713 PMC2667857 19299678

[29] Centre for Infectious Disease Research in Zambia. Cervical Cancer Prevention Program in Zambia. Training Manual For Peer Education. Lusaka: CIDR; 2009. p. 1–42.

[30] IBM Software: SPSS Statistics Base: Features. http://www-03.ibm.com/software/products/en/spss-stats-base. Accessed 15 May 2017.

[31] Trochim WM, Donnelly JP (2001). Research methods knowledge base.

[32] Ndikom CM, Ofi BA (2012). Awareness, perception and factors affecting utilization of cervical cancer screening services among women in Ibadan, Nigeria: a qualitative study. Reprod Health.

[33] Chadza E, Chirwa E, Maluwa A, Kazembe A, Chimwaza A (2012). Factors that contribute to delay in seeking cervical cancer diagnosis and treatment among women in Malawi.

[34] Shakibazadeh E, Ahmadnia E, Akbari F, Negarandeh R (2009). Barriers and motivating factors related to cervical cancer screening. HAYAT.

[35] Ntemana TJ, Olatokun W (2012). Analyzing the Influence of Diffusion of Innovation Attributes on Lecturers’ Attitude Towards Information and Communication Technologies.

[36] Communication for Governance & Accountability Program. Theories of Behavior Change: The World Bank. http://documents.worldbank.org/curated/en/456261468164982535/Theories-of-behavior-change. Accessed 15 May 2017.

[37] Hong L, Robertson J (2011). Catanzarite J.

[38] Sloane BC, Zimmer CG (1993). The power of peer health education. J Am Coll Heal.

[39] Nguyen LT, Tran BX, Tran CT, Le HT, Tran SV (2014). The cost of antiretroviral treatment service for patients with HIV/AIDS in a central outpatient clinic in Vietnam. Clinicoecon Outcomes Res.

[40] Mataria A, Giacaman R, Khatib R, Moatti J-P (2006). Impoverishment and patients’ “willingness” and “ability” to pay for improving the quality of health care in Palestine: an assessment using the contingent valuation method. Health Policy.

