# Supplementary material for: Effects of peer health education on perception and practice of screening for cervical cancer among urban residential women in south-east Nigeria: a before and after study
Source: BMC Womens Health. 2017 Jun 9;17:41. doi: 10.1186/s12905-017-0399-6 (PMC5466744; doi:10.1186/s12905-017-0399-6)
Supplement: Additional file 1: — Questionnaire for data collection on knowledge, perception and practice of screening for cervical cancer. Structured questionnaire with two open-ended questions, and having three sections namely, (i) personal data, (ii) knowledge and perception of cervical cancer and cervical cancer screening, (iii) utilization of cervical cancer screening services and determinants. (PDF 337 kb) [file 12905_2017_399_MOESM1_ESM.pdf]

APPENDIX: QUESTIONNAIRE FOR DATA COLLECTION ON KNOWLEDGE, PERCEPTION AND PRACTICE OF SCREENING FOR CERVICAL CANCER

SECTION A: PERSONAL DATA

Mark (√) in the spaces provided, where appropriate. Only one tick is required per question.
Research Identification number:- ………………..

1. Age at last birthday ………………………
2. Marital status
   a. Married [ ]
   b. Never married [ ]
   c. Divorced/Separated [ ]
   d. Widowed [ ]
   e. Living with partner/Co-habiting [ ]
3. Tribe
   a. Igbo [ ]
   b. Yoruba [ ]
   c. Hausa [ ]
   d. Other, please specify …………………………………
4. Religion
   a. Christianity [ ]
   b. Islam [ ]
   c. Traditional [ ]
5. Highest level of education
   a. No formal education [ ]
   b. Primary school: completed [ ] Not completed [ ]
   c. Secondary school: completed [ ] Not completed [ ]
   d. Tertiary school: completed [ ] Not completed [ ]
6. Employment status
   a. Employed [ ]
   b. Self employed [ ]
   c. Unemployed [ ]
   d. Student [ ]
7. Number of children
   a. One [ ]
   b. Two [ ]
   c. Three [ ]
   d. Four [ ]
   e. Above four [ ]

SECTION C: PERCEPTION AND ATTITUDE TO CERVICAL CANCER AND CERVICAL CANCER SCREENING

1. Have you ever heard of cervical cancer or cancer of the cervix?
   Yes [ ] No [ ]
2. How serious is cervical cancer compared to other forms of cancer
   a. More severe than other cancers [ ]
   b. Same as other cancers [ ]
   c. Less severe than other cancers [ ]
   d. Don't know [ ]
3. Is cancer of the cervix curable?
   a. Never [ ]

b. Yes, if detected early [ ]
c. Always [ ]
d. Don't know [ ]

4. Is there any possibility that you could have cancer of the cervix?
   a. Yes [ ]
   b. Maybe [ ]
   c. No [ ]
   d. Don't know [ ]
5. How would you judge your risk of developing cervical cancer?
   a. High risk [ ]
   b. Low risk [ ]
   c. No risk [ ]
   d. Don't know [ ]
6. What do you think about screening for cervical cancer?
   a. It is not important [ ]
   b. It is very important [ ]
   c. It is important [ ]
   d. I feel indifferent about it [ ]
   e. I don't have any opinion about it [ ]
7. What do you think about screening regularly for cervical cancer?
   a. It is always necessary [ ]
   b. It is only necessary if there was a bad result [ ]
   c. It is not necessary [ ]
   d. I feel indifferent about it
   e. I don't have any opinion about it [ ]
8. What are the benefits of screening for cervical cancer? (*you can choose more than one answer)*
   a. It can detect cervical cancer early [ ]
   b. It gives me a sense of control [ ]
   c. There are no benefits [ ]
   d. I don't know of any benefits [ ]
   e. Others *(please specify)* ……………………………………………………………………………………….
9. Would you accept to screen for cervical cancer if the services are available?
   a. Yes [ ]
   b. No [ ]
   c. Maybe [ ]
   d. Never [ ]
10. If you ticked "Maybe", what conditions would make you accept it or not accept it?

……
…………………………………

SECTION D: UTILIZATION OF CERVICAL CANCER SCREENING SERVICES AND DETERMINANTS

1. Have you ever screened for cervical cancer?
   a. Yes [ ]
   b. No [ ]

If you answered No to question 1, move to question 6. If Yes, continue with question 2.

2. When was the last time you screened for cervical cancer?
   a. < 1 year [ ]
   b. Over a year ago, but not up to 3 years ago [ ]
   c. More than 3 years ago [ ]

    d. More than 10 years ago [ ]
    e. Can't remember [ ]
3. How often do you screen for cervical cancer?
    a. Regularly, every 1-3 years [ ]
    b. Anytime I remember [ ]
    c. Whenever I hear someone died of cancer [ ]
    d. Whenever my doctor says so [ ]
    e. Don't know [ ]
    f. Others, *(please specify)*…………………………………………………………………
4. What method did you use the last time you screened for cervical cancer?
    a. Pap smear test [ ]
    b. Visual Inspection with Lugol's Iodine (VILI) [ ]
    c. Visual Inspection with Acetic Acid (VIA) [ ]
    d. Don't know the name [ ]
    e. Others, *(please specify)* …………………………………………………………………
5. Why do you screen for cervical cancer? (*you can choose more than one answer)*
    a. I can afford the cost of screening [ ]
    b. Because if it is detected early, it can be cured [ ]
    c. Because I could have the disease [ ]
    d. Because my doctor recommends it [ ]
    e. Because cervical cancer kills and I don't want to die from it [ ]
    f. My family encourages me too [ ]
    g. My friend died of cervical cancer [ ]
    h. A close relation died of cancer [ ]
    i. Others *(please specify)* ……………………………………………………………………
6. Why don't you screen for cervical cancer? (*you can choose more than one answer)*
    a. I don't know any screening tests [ ]
    b. Because I can't have cervical cancer [ ]
    c. I am scared of receiving a bad result [ ]
    d. I don't have any symptoms [ ]
    e. It's a waste of money [ ]
    f. It is against my faith [ ]
    g. It is expensive [ ]
    h. There is no screening center near my house [ ]
    i. Our culture forbids it [ ]
    j. I will become infertile [ ]
    k. I hear it is painful [ ]
    l. Others *(please specify)* ……………………………………………………………………
7. Could you suggest ways of improving women's knowledge of cervical cancer and screening for cervical cancer.
    ………………………………………………………………………………………….
    ………………………………………………………………………………………………………
    ………………………………………………………………………………………………………
    ………………………………………………………………………………………………………
